# Supplementary material for: Transform[ing] heart failure professionals with Indigenous land-based cultural safety in Ontario, Canada
Source: PLoS One. 2024 May 23;19(5):e0302816. doi: 10.1371/journal.pone.0302816 (PMC11115247; doi:10.1371/journal.pone.0302816)
Supplement: Attachment — Submitted filename: Response to Reviewers Transforming.docx [file pone.0302816.s001.docx]

Response to Reviewers: Transform[ing] heart failure professionals with Indigenous land-based cultural safety in Ontario, Canada

Dear academic editor and reviewers,

Thank you for your thorough review of our article, titled “Transform[ing] heart failure professionals with Indigenous land-based cultural safety in Ontario, Canada”. In the following rebuttal letter we have included each point raised by the academic editor and reviewers, followed by our rebuttal in red font. We have also attached a marked-up copy of the manuscript that highlights changes made to the original version and an unmarked version without tracked changes.

Journal requirements:

1. When submitting your revision, we need you to address these additional requirements.

Please ensure that your manuscript meets PLOS ONE's style requirements, including those for file naming. The PLOS ONE style templates can be found at 
[https://journals.plos.org/plosone/s/file?id=wjVg/PLOSOne_formatting_sample_main_body.pdf](https://urldefense.com/v3/__https:/journals.plos.org/plosone/s/file?id=wjVg*PLOSOne_formatting_sample_main_body.pdf__;Lw!!CjcC7IQ!M0do89frCs2UffoSj1akX-__LfM4LQdcy7Ucc_ldqRxZlecDGBtMKvm8QfTwXbKCK7fHNgceiFS6yH5CNysTz6TfRe7FBRg$) and 
[https://journals.plos.org/plosone/s/file?id=ba62/PLOSOne_formatting_sample_title_authors_affiliations.pdf](https://urldefense.com/v3/__https:/journals.plos.org/plosone/s/file?id=ba62*PLOSOne_formatting_sample_title_authors_affiliations.pdf__;Lw!!CjcC7IQ!M0do89frCs2UffoSj1akX-__LfM4LQdcy7Ucc_ldqRxZlecDGBtMKvm8QfTwXbKCK7fHNgceiFS6yH5CNysTz6TfZRWI88g$).

Updated manuscript body text to meet formatting requirements of Level 1 and Level 2 headings, including bold type, 18pt/16pt font, and sentence case.

Corrected figure citations from Figures 1a-i and 2a-c to Fig 1-12, and used “Fig X” format for in-text citations, figure titles, and file names. Moved figure titles to directly after the paragraph in which they are first mentioned. Reformatted figures from .png to .tiff files, within dpi, height, width, and KB limits and adjusted in-figure text to Arial size 8-12, removed figure titles and rendered in grayscale.

Edited in-text citations to use square brackets rather than rounded brackets.

On title page, title was centered and moved to start at line 4. Short title was removed as it was not identified within the PLOS ONE formatting guidelines.

Author affiliations were changed from letters to numbers. Affiliation street addresses and postal codes were removed, provinces were written in full, periods were removed at the end of the address lines. Co-author email addresses and ORCIDs were removed. Spaces were removed between affiliation superscripts and author names. Authors names were collapsed into one sentence from separate lines. Four spaces were added after the authors names, and affiliation addresses were moved to directly below the author names.

Corresponding author was indicated with a superscript asterix and other corresponding author details were removed, with the exception of an email address. Corresponding author initials were indicated after the email address in parathenses.

Contributorship was changed to be indicated by the symbols provided.

2. Note from Emily Chenette, Editor in Chief of PLOS ONE, and Iain Hrynaszkiewicz, Director of Open Research Solutions at PLOS: Did you know that depositing data in a repository is associated with up to a 25% citation advantage ([https://doi.org/10.1371/journal.pone.0230416)?](https://urldefense.com/v3/__https:/doi.org/10.1371/journal.pone.0230416)?__;!!CjcC7IQ!M0do89frCs2UffoSj1akX-__LfM4LQdcy7Ucc_ldqRxZlecDGBtMKvm8QfTwXbKCK7fHNgceiFS6yH5CNysTz6Tf6Cqkyzk$) If you’ve not already done so, consider depositing your raw data in a repository to ensure your work is read, appreciated and cited by the largest possible audience. You’ll also earn an Accessible Data icon on your published paper if you deposit your data in any participating repository ([https://plos.org/open-science/open-data/#accessible-data](https://urldefense.com/v3/__https:/plos.org/open-science/open-data/*accessible-data__;Iw!!CjcC7IQ!M0do89frCs2UffoSj1akX-__LfM4LQdcy7Ucc_ldqRxZlecDGBtMKvm8QfTwXbKCK7fHNgceiFS6yH5CNysTz6TfJVjrLlY$)).

3. We note that the grant information you provided in the ‘Funding Information’ and ‘Financial Disclosure’ sections do not match.

When you resubmit, please ensure that you provide the correct grant numbers for the awards you received for your study in the ‘Funding Information’ section.

The correct funding information/financial disclosure is as follows: “The On-the-Land Indigenous Education Program was funded by 2021 TRANSFORM HF Collaboration Starter Grant awarded to the P.I., Dr. Mashford-Pringle. TRANSFORM HF is funded by the Ted Rogers Centre for Heart Research and the University of Toronto [see https://transformhf.ca/]. The funders had no role in study design, data collection and analysis, decision to publish, or preparation of the manuscript.”

There is no grant number as TRANSFORM HF is held by the University Health Network, which is also affiliated with the University of Toronto, where Dr. Mashford-Pringle’s lab is held. As a result, the money is transferred between institutions without the need for a grant agreement.

4. Thank you for stating the following financial disclosure: 
 [The On-the-Land Indigenous Education Program was funded by 2021 TRANSFORM HF Collaboration Starter Grant awarded to the P.I., Dr. Mashford-Pringle. TRANSFORM HF is funded by the Ted Rogers Centre for Heart Research and the University of Toronto [see [https://transformhf.ca/]].](https://urldefense.com/v3/__https:/transformhf.ca/*5D*5D.__;JSU!!CjcC7IQ!M0do89frCs2UffoSj1akX-__LfM4LQdcy7Ucc_ldqRxZlecDGBtMKvm8QfTwXbKCK7fHNgceiFS6yH5CNysTz6TfNUqFdlA$)  
Please state what role the funders took in the study.  If the funders had no role, please state: ""The funders had no role in study design, data collection and analysis, decision to publish, or preparation of the manuscript."" 
If this statement is not correct you must amend it as needed. 
Please include this amended Role of Funder statement in your cover letter; we will change the online submission form on your behalf.

“The funders had no role in study design, data collection and analysis, decision to publish, or preparation of the manuscript.” – This statement is true. Thank you for adding it on our behalf.

5. We note that you have indicated that there are restrictions to data sharing for this study. PLOS only allows data to be available upon request if there are legal or ethical restrictions on sharing data publicly. For more information on unacceptable data access restrictions, please see [http://journals.plos.org/plosone/s/data-availability#loc-unacceptable-data-access-restrictions.](https://urldefense.com/v3/__http:/journals.plos.org/plosone/s/data-availability*loc-unacceptable-data-access-restrictions.__;Iw!!CjcC7IQ!M0do89frCs2UffoSj1akX-__LfM4LQdcy7Ucc_ldqRxZlecDGBtMKvm8QfTwXbKCK7fHNgceiFS6yH5CNysTz6TfkiwKAFM$)

Before we proceed with your manuscript, please address the following prompts:

a) If there are ethical or legal restrictions on sharing a de-identified data set, please explain them in detail (e.g., data contain potentially identifying or sensitive patient information, data are owned by a third-party organization, etc.) and who has imposed them (e.g., a Research Ethics Committee or Institutional Review Board, etc.). Please also provide contact information for a data access committee, ethics committee, or other institutional body to which data requests may be sent.

Data cannot be shared publicly because there may be some identifiable phrases in the talking circle data. Open text answers to the survey questions and talking circle data contain detailed recounting of personal life stories and experiences, which contains potentially identifying information, especially given the small sample size.

Data are available from the University of Toronto Research Ethics Committee (contact Office of Research Ethics, University of Toronto, McMurrich Building, 2nd floor, 12 Queen’s Park Crescent West, Toronto, ON M5S 1S8 416-946-3273, ethics.review@utoronto.ca) for researchers who meet the criteria for access to confidential data

b) If there are no restrictions, please upload the minimal anonymized data set necessary to replicate your study findings to a stable, public repository and provide us with the relevant URLs, DOIs, or accession numbers. For a list of recommended repositories, please see
[https://journals.plos.org/plosone/s/recommended-repositories](https://urldefense.com/v3/__https:/journals.plos.org/plosone/s/recommended-repositories__;!!CjcC7IQ!M0do89frCs2UffoSj1akX-__LfM4LQdcy7Ucc_ldqRxZlecDGBtMKvm8QfTwXbKCK7fHNgceiFS6yH5CNysTz6Tf_2wapQA$). You also have the option of uploading the data as Supporting Information files, but we would recommend depositing data directly to a data repository if possible.

We will update your Data Availability statement on your behalf to reflect the information you provide.

6. Please include captions for your Supporting Information files at the end of your manuscript, and update any in-text citations to match accordingly. Please see our Supporting Information guidelines for more information: [http://journals.plos.org/plosone/s/supporting-information.](https://urldefense.com/v3/__http:/journals.plos.org/plosone/s/supporting-information.__;!!CjcC7IQ!M0do89frCs2UffoSj1akX-__LfM4LQdcy7Ucc_ldqRxZlecDGBtMKvm8QfTwXbKCK7fHNgceiFS6yH5CNysTz6TfHTENZcQ$)

No Supporting Information files were included.

7. Please review your reference list to ensure that it is complete and correct. If you have cited papers that have been retracted, please include the rationale for doing so in the manuscript text, or remove these references and replace them with relevant current references. Any changes to the reference list should be mentioned in the rebuttal letter that accompanies your revised manuscript. If you need to cite a retracted article, indicate the article’s retracted status in the References list and also include a citation and full reference for the retraction notice.

The reference list was reviewed and found to be complete and correct.

Additional Editor Comments:

It is recommended that the authors take into account the six observations made by the reviewers. This is an adjustment to the article that can improve its quality and make it more coherent according to their perspective of working with an indigenous population.

The manuscript presents a very interesting case for Indigenous land-based learning. I think the topic is worth being published but I suggest some major improvements before acceptance. This paper has a relevant theme, namely, the relationship between health and cultural practices. It analyzes the participants' impressions of the importance of the learning program described in the text. The approach to the results favors quantitative data, although there is no statistical treatment, since the sample size does not allow such an analysis.

1. I suggest an analysis of the discourse of health professionals on issues related to the specific health of indigenous people before and after the training. I think it would be a rich form of analysis and would complete the article.

We appreciate the depth this would add to the analysis but unfortunately this is not a topic we examined in-depth within this study.

2. In this way they would have elements to elaborate conclusions in a more conducive and more articulated way with a cultural epistemology and not from an external point of view.

This appears to be a follow-up sentence to the piece above.

3. A few more words on the Indigenous land-based approach, for the reader who is not familiar

Two additional sentences were added to the introduction to detail Indigenous land-based learning.

4. Bit more details about participants background, about the learning activities

Participants were trainees (Master, PhD and Post-Doctoral), clinicians (physicians and nurses), and researchers. The details in brackets have been added to the methods section.

The learning activities were comprised of land-based knowledges provided by Elders and Knowledge Keepers as indicated in the methods section previously. We cannot provide a detailed description as this is the First Nations Elders and Knowledge Keepers knowledge which can only be stated by them. The discussions held by the PI, have not been released as this part of the project has not been written about yet and it would be pre-emptive to state all the topics covered.

5. The pre post surverys descriptive statistics are not particularly interesting or informative. I understand that in the attempt to evaluate the method, it is necessary to do some surveys. However the most important part would be how participants reported their experience qualitatively. My suggestion is to sum up the descriptives in just 1 or 2 graphs max (by the way all the necessary information must be provided in the graphs, such as N=). Then discuss for instance how the medical or enginneeer epistemological framework negotiated with the indigenous one. Medical approach is a postivist, causalitic understandning. How did they really interact with the indigenous epistemology/cosmology? Or are you just looking for a behavioral effect? Just doing things differently when dealing with first nations?

Thank you so much for your comment. We did not evaluate whether positivistic or Indigenous epistemologies were different or if participants understood the differences between them. We have provided the self-reported change in knowledge and any changes in behaviour that was provided by the participants. Our graphs have been reduced to sum up the most important data found in the survey analysis.

6. Conclusions are a bit weak and leave a sense of "so what"? can be elaborated

Conclusion section was edited to clarify and strengthen the sense of “so what”.

[Note: HTML markup is below. Please do not edit.]

Reviewers' comments:

Reviewer's Responses to Questions

**Comments to the Author**

1. Is the manuscript technically sound, and do the data support the conclusions?

The manuscript must describe a technically sound piece of scientific research with data that supports the conclusions. Experiments must have been conducted rigorously, with appropriate controls, replication, and sample sizes. The conclusions must be drawn appropriately based on the data presented.

Reviewer #1: Partly

Reviewer #2: Partly

Reviewer comments are addressed in the appropriate sections.

2. Has the statistical analysis been performed appropriately and rigorously?

Reviewer #1: N/A

Reviewer #2: N/A

No action was required.

3. Have the authors made all data underlying the findings in their manuscript fully available?

The [PLOS Data policy](https://urldefense.com/v3/__http:/www.plosone.org/static/policies.action*sharing__;Iw!!CjcC7IQ!M0do89frCs2UffoSj1akX-__LfM4LQdcy7Ucc_ldqRxZlecDGBtMKvm8QfTwXbKCK7fHNgceiFS6yH5CNysTz6Tf9UzBvqA$) requires authors to make all data underlying the findings described in their manuscript fully available without restriction, with rare exception (please refer to the Data Availability Statement in the manuscript PDF file). The data should be provided as part of the manuscript or its supporting information, or deposited to a public repository. For example, in addition to summary statistics, the data points behind means, medians and variance measures should be available. If there are restrictions on publicly sharing data—e.g. participant privacy or use of data from a third party—those must be specified.

Reviewer #1: No

Reviewer #2: Yes

The data availability statement was updated to include further reasoning as to why the data cannot be shared publically.

4. Is the manuscript presented in an intelligible fashion and written in standard English?

PLOS ONE does not copyedit accepted manuscripts, so the language in submitted articles must be clear, correct, and unambiguous. Any typographical or grammatical errors should be corrected at revision, so please note any specific errors here.

Reviewer #1: Yes

Reviewer #2: Yes

No action was required.

5. Review Comments to the Author

Please use the space provided to explain your answers to the questions above. You may also include additional comments for the author, including concerns about dual publication, research ethics, or publication ethics. (Please upload your review as an attachment if it exceeds 20,000 characters)

Reviewer #1: The manuscript presents a very interesting case for Indigenous land-based learning. I think the topic is worth being published but I suggest some major improvements before acceptance.
1) A few more words on the Indigenous land-based approach, for the reader who is not familiar

Two additional sentences were added to the introduction to detail Indigenous land-based learning.

2) Bit more details about participants background, about the learning activities

Participants were trainees (Master, PhD and Post-Doctoral), clinicians (physicians and nurses), and researchers. The details in brackets have been added to the methods section.

The learning activities were comprised of land-based knowledges provided by Elders and Knowledge Keepers as indicated in the methods section previously. We cannot provide a detailed description as this is the First Nations Elders and Knowledge Keepers knowledge which can only be stated by them. The discussions held by the PI, have not been released as this part of the project has not been written about yet and it would be pre-emptive to state all the topics covered.

3) The pre post surverys descriptive statistics are not particularly interesting or informative. I understand that in the attempt to evaluate the method, it is necessary to do some surveys. However the most important part would be how participants reported their experience qualitatively. My suggestion is to sum up the descriptives in just 1 or 2 graphs max (by the way all the necessary information must be provided in the graphs, such as N=). Then discuss for instance how the medical or enginneeer epistemological framework negotiated with the indigenous one. Medical approach is a postivist, causalitic understandning. How did they really interact with the indigenous epistemology/cosmology? Or are you just looking for a behavioral effect? Just doing things differently when dealing with first nations?

Thank you so much for your comment. We did not evaluate whether positivistic or Indigenous epistemologies were different or if participants understood the differences between them. We have provided the self-reported change in knowledge and any changes in behaviour that was provided by the participants. Our graphs have been reduced to sum up the most important data found in the survey analysis.

4) what is the understanding of hearth deseases in indigenous medicine?

This is not what this article was designed to explore. We were studying the impacts of land-based learning and cultural safety among those who work in heart health research, primary care, and biotech in Toronto, Ontario, Canada.

5) conclusions are a bit weak and leave a sense of "so what"? can be elaborated

Conclusion section was edited to clarify and strengthen the sense of “so what”.

Reviewer #2: This work has a relevant subject, i.e., the relations between health and cultural practices. It analyzes the impressions of the participants about the importance of the learning program that was described in the text. The approach of the results privileges the quantitative data, even that it hasn't statistical treatment, because the size of the sample doesn't for an analyzis such that. Personally - and it says about my formation as a qualitative researcher - I would be more interested in the discourse of health professionals on issues involving the specific health of indigenous people before and after the training. I think that this would be a rich way of analyzis (that the authors may explore in other studies). As far as this study is concerned, I can find no reason to reject it. On the contrary, the relevance of the topic is adequately justified and the conclusions are based on the results described.

Thank you for this review. The discourse comment has been noted but is not a topic we examined in-depth within this study.

6. PLOS authors have the option to publish the peer review history of their article ([what does this mean?](https://urldefense.com/v3/__https:/journals.plos.org/plosone/s/editorial-and-peer-review-process*loc-peer-review-history__;Iw!!CjcC7IQ!M0do89frCs2UffoSj1akX-__LfM4LQdcy7Ucc_ldqRxZlecDGBtMKvm8QfTwXbKCK7fHNgceiFS6yH5CNysTz6Tf_vfX7Hw$)). If published, this will include your full peer review and any attached files.


If you choose “no”, your identity will remain anonymous but your review may still be made public.


**Do you want your identity to be public for this peer review?** For information about this choice, including consent withdrawal, please see our [Privacy Policy](https://urldefense.com/v3/__https:/www.plos.org/privacy-policy__;!!CjcC7IQ!M0do89frCs2UffoSj1akX-__LfM4LQdcy7Ucc_ldqRxZlecDGBtMKvm8QfTwXbKCK7fHNgceiFS6yH5CNysTz6TfsglUPT0$).

Reviewer #1: No

Reviewer #2: **Yes:** Filipe Degani-Carneiro

No action was required.

[NOTE: If reviewer comments were submitted as an attachment file, they will be attached to this email and accessible via the submission site. Please log into your account, locate the manuscript record, and check for the action link "View Attachments". If this link does not appear, there are no attachment files.]

While revising your submission, please upload your figure files to the Preflight Analysis and Conversion Engine (PACE) digital diagnostic tool, [https://pacev2.apexcovantage.com/](https://urldefense.com/v3/__https:/pacev2.apexcovantage.com/__;!!CjcC7IQ!M0do89frCs2UffoSj1akX-__LfM4LQdcy7Ucc_ldqRxZlecDGBtMKvm8QfTwXbKCK7fHNgceiFS6yH5CNysTz6Tfrwgp9qw$). PACE helps ensure that figures meet PLOS requirements. To use PACE, you must first register as a user. Registration is free. Then, login and navigate to the UPLOAD tab, where you will find detailed instructions on how to use the tool. If you encounter any issues or have any questions when using PACE, please email PLOS at [figures@plos.org](mailto:figures@plos.org). Please note that Supporting Information files do not need this step.
